# A pan-cancer analysis of the oncogenic and immunological roles of transglutaminase 1 (TGM1) in human cancer

**DOI:** 10.1007/s00432-024-05640-6

**Published:** 2024-03-12

**Authors:** Ruicheng Wu, Dengxiong Li, Shuxia Zhang, Jie Wang, Kai Chen, Zhouting Tuo, Akira Miyamoto, Koo Han Yoo, Wuran Wei, Chi Zhang, Dechao Feng, Ping Han

**Affiliations:** 1 Department of Urology, Institute of Urology, West China School of Medicine, West China Hospital, Sichuan University, Chengdu, 610041 China; 2 Research Core Facilities, West China Hospital, Sichuan University, Chengdu, 610041 Sichuan People’s Republic of China; 3 Department of Urology, The Second Affiliated Hospital of Anhui Medical University, Hefei, 230601 China; 4 Department of Rehabilitation, West Kyushu University, Fukuoka, Japan; 5 Department of Urology, Kyung Hee University, Seoul, South Korea; 6 Department of Rehabilitation, The Affiliated Hospital of Southwest Medical University, Luzhou, 646000 People’s Republic of China

**Keywords:** Pan-cancer biomarker, Tumor-infiltrating cells, Transglutaminase 1

## Abstract

**Background:**

There is currently a limited number of studies on transglutaminase type 1 (TGM1) in tumors. The objective of this study is to perform a comprehensive analysis across various types of cancer to determine the prognostic significance of TGM1 in tumors and investigate its role in the immune environment.

**Method:**

Pan-cancer and mutational data were retrieved from the TCGA database and analyzed using R (version 3.6.4) and its associated software package. The expression difference and prognosis of TGM1 were examined, along with its correlation with tumor heterogeneity, stemness, mutation landscape, and RNA modification. Additionally, the relationship between TGM1 expression and tumor immunity was investigated using the TIMER method.

**Results:**

TGM1 is expressed differently in various tumors and normal samples and is associated with the overall survival and progression-free time of KIRC, ACC, SKCM, LIHC, and STES. In LICH, we found a negative correlation between TGM1 expression and 6 indicators of tumor stemness. The mutation frequencies of BLCA, LIHC, and KIRC were 1.7%, 0.3%, and 0.3% respectively. In BLCA and BRCA, there was a significant correlation between TGM1 expression and the infiltration of CD4 + T cells, CD8 + T cells, neutrophils, and dendritic cells.

**Conclusion:**

TGM1 has the potential to serve as both a prognostic marker and a drug target.

**Supplementary Information:**

The online version contains supplementary material available at 10.1007/s00432-024-05640-6.

## Introduction

With the increase in population aging, the burden of disease on health is expected to become increasingly heavy (Zhang et al. 2023). Statistics indicate that the likelihood of cancer in individuals aged 65 and above is ten times higher compared to those under 65, and the aging process is closely linked to tumorigenesis (Wang et al. 2022). Advanced age is associated with several cancer risk factors, including prostate and bladder cancer (Feng et al. 2022a; Jin et al. 2023). Consequently, as the global population continues to age, malignant tumors are poised to become increasingly significant threats to human life and well-being. This scenario presents a formidable challenge to global healthcare (Siegel et al. 2023; Yan et al. 2022). While current treatment methods have undoubtedly improved the prognosis for cancer patients, there remains a subset of patients who exhibit poor response to treatment, along with instances of metastasis and recurrence, ultimately leading to low survival rates (Klein 2020; Phillips 2022). Therefore, it is imperative to quest new markers for the diagnosis and treatment of tumors.

Previous studies have primarily focused on transglutaminase type 2 (TGM2) within the TGM family, which has been associated with tumor proliferation, invasion, and drug resistance (Lee et al. 2018; Tempest, et al. 2021; Eckert et al. 2015).The enzyme TGM2 encodes the catalysis of cross-linking between glutamine and lysine residues, which plays a crucial role in stabilizing the extracellular matrix, cytoskeletal function, adhesion, and signaling (Bianchi et al. 2018). The conformation of TGM2 is regulated by cellular stimuli, such as changes in calcium levels, resulting in different biological effects (Lai and Greenberg 2013). Several studies have shown that TGM2 exhibits strong expression and activity in the stromal tissue surrounding tumors, indicating its involvement in tumor aggressiveness (Malkomes et al. 2023; Zhang et al. 2023). Due to its diverse functions and implications in disease, TGM2 has emerged as a potential therapeutic target. However, there has been limited research on transglutaminase type 1 (TGM1) in relation to tumors (Zhong et al. 2019). TGM1 plays a crucial role in the formation of the cornified cell envelope and is essential for maintaining skin barrier function (Boeshans et al. 2007). The potential of TGM1 in tumors remains largely unexplored, and there is a lack of comprehensive studies investigating the correlation between TGM1 expression and various types of tumors. To address this gap, we conducted a pan-cancer analysis using data from The Cancer Genome Atlas (TCGA). Our findings demonstrate that TGM1 exhibits prognostic value across a range of tumors and holds promise as a potential biomarker.

## Methods

### Date acquisition and differential and analysis

Consistent with our previous study (Feng et al. 2022b; Shi et al. 2023), we obtained the TCGA pan-cancer dataset from the USCS database ( (Goldman et al. 2020). In addition, we integrated the TCGA prognostic dataset from previous study and extracted the expression data of TGM1 in each sample (Liu et al. 2018). Samples were screened for an expression level of 0, starting from Solid Tissue Normal, Primary tumor and Primary Blood Derived Cancer-Peripheral Blood. Subsequently, a log2 (*x* + 0.001) transformation was performed on each expression quality, and cancer types with a sample size of less than 10 were eliminated, and the unpaired Wilconxon rank sum test and sign test were used for significant difference analysis.

### Pan-cancer survival analysis and relationship with clinical features

We screened metastatic samples from Primary Blood Derived Cancer-Peripheral Blood (TCGA-LAML), Primary Tumor, and TCGA-SKCM databases. After excluding samples with an expression level of 0 or a follow-up time of less than 30 days, we obtained expression data, overall survival (OS), and progression-free interval (PFI) data for 39 cancer types. The data were analyzed using a Cox proportional hazards regression model and the log-rank test was used to obtain prognostic significance (Andersen and Gill 1982). Furthermore, the unpaired Wilcoxon rank sum test, sign test, and Kruskal test were utilized to evaluate the correlation between TGM1 expression and clinical stage, gender, and other clinical characteristics. In addition, we investigated the correlation between TGM1 mRNA expression and the age of the patients. We utilized proportional hazards hypothesis testing and Cox regression analysis to investigate various urological tumors. Subsequently, we conducted nomogram analysis and visualization.

### Analysis of tumor heterogeneity, stemness and gene mutation

We calculated tumor stemness indicators by analyzing tumor methylation and mRNA expression signatures. This included six indexes namely DNA methylation based (DNAss), differentially methylated probes-based (DMPss), enhancer elements/DNA methylation-based (ENHss), RNA expression-based (RNAss), epigenetically regulated DNA methylation-based (EREG-METHss) and epigenetically regulated RNA methylation-based (EREG-METHss). Additionally, we conducted Spearman analysis to determine the correlation between tumor stemness characteristics and TGM1 expression. Tumor mutation burden (TMB), mutant-allele tumor heterogeneity (MATH), tumor ploidy, tumor purity, loss of heterozygosity (LOH), neoantigen (NEO), and homologous recombination deficiency (HRD) are indicators that reflect tumor heterogeneity (Ozga et al. 2021). These indicators were obtained from GDC (https://protal.gdc.cancer.gov/) and processed using Mutcet2 software and the R package 'maftools’ (Beroukhim et al. 2010), and microsatellite instability (MSI) was analyzed to examine the relationship between TMG1 expression and tumor heterogeneity, using the Spearman rank correlation coefficient (Bonneville et al. 2017). The Mutect2 software was utilized to process a simple nucleotide variation dataset for the analysis of gene mutations. Following the integration of the data, gene expression and mutations were detected in ACC, BLCA, KIRC, and LIHC. To evaluate the difference in mutation frequency among each group of samples, the chi-square test was employed.

### Analysis of RNA modifications, tumor immune microenvironment and drug sensitivity

We conducted an analysis to examine the correlation between TGM1 mRNA expression levels and a total of 36 stimulatory and 24 heterogeneous checkpoints (Ozga et al. 2021), as well as 150 immune regulatory genes (receptor, MHC, chemokine, immunoinhibitory, immunostimulator (Shen et al. 2011)). The correlation of TGM1 expression with 44 genes in three RNA modification types was analyzed using data matrix, including 10 m1A genes, 13 m5C genes and 21 m6A genes. The tumor microenvironment was assessed using Timer (Li et al. 2017), a tool available in the R package 'IOBR' (Ru et al. 2019). Furthermore, we investigated the Genomics of Drug Sensitivity in Cancer (GDSC) and the Cancer Therapeutics Response Portal (CTRP) drug sensitivity in pan-cancer via GSCALite (Liu et al. 2018). In addition, we mapped the gene expression profiles of tumors to GeneSymbol. To assess the stromal scores of each patient in each tumor, we utilized the R software package ESTIMATE and calculated them based on gene expression (Yoshihara et al. 2013).

### Statistical analysis

All the analysis were performed with the use of R software (version 3.6.3) and its suitable packages. A p-value below 0.05 was considered statistical significance. The Wilcoxon rank sum test and sign test were utilized to analyze paired differences, while the Kruskal test was employed to test multiple groups of samples. Correlation analysis between two variables was conducted using the Spearman test.

## Results

### Differential expression and clinical characteristics of TGM1

**Fig. 1:**
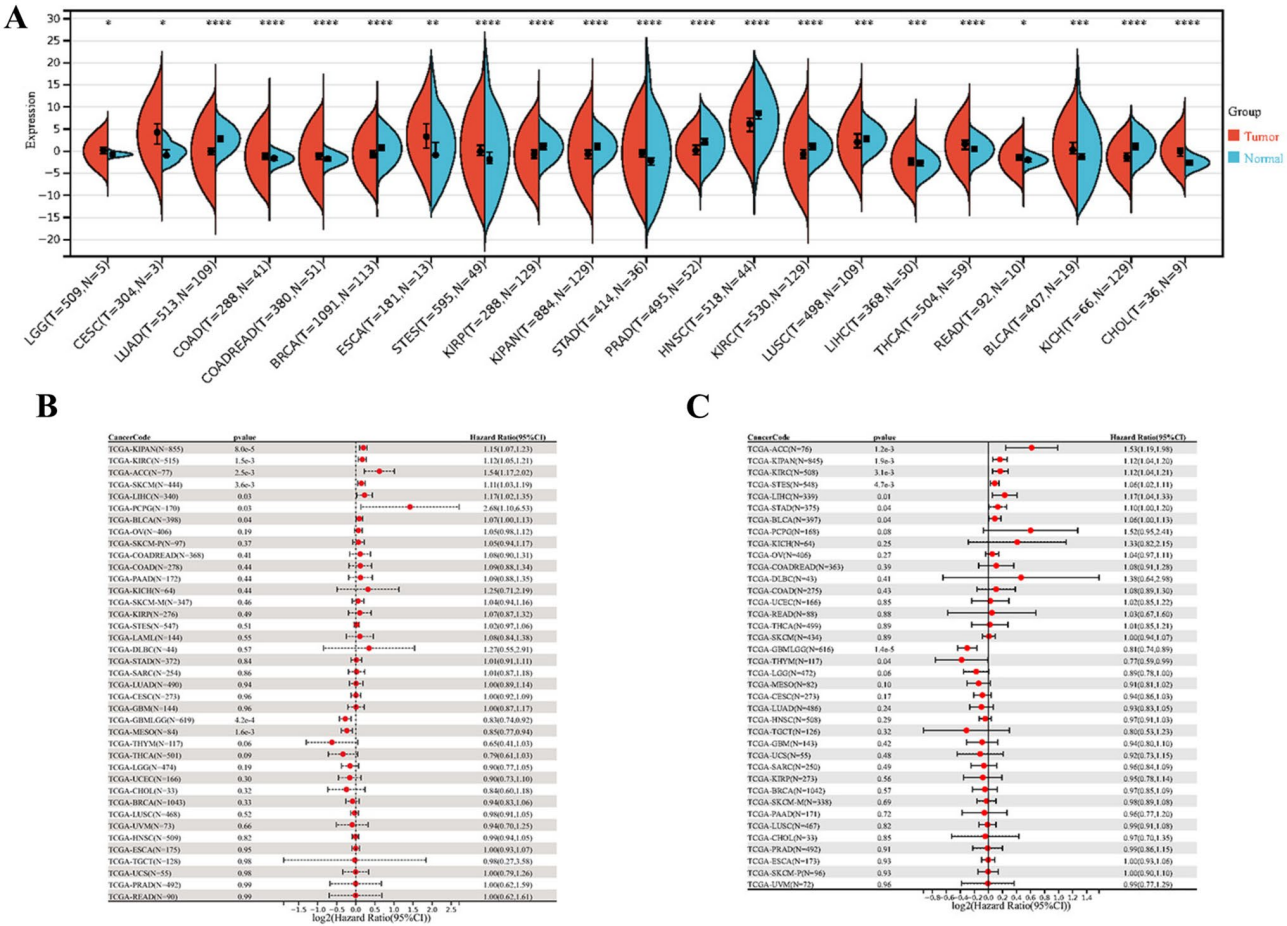
Differential expression and prognosis analysis of TGM1. **A** pan-cancer analysis of TGM1 for differential expression between tumor and normal tissues; **B** pan-cancer analysis of TGM1 for OS;** C** pan-cancer analysis of TGM1 for PFI; OS: overall survival; PFI: progression-free interval

Compared to normal samples, we found that the TGM1 mRNA expression was significantly upregulated in Cervical squamous cell carcinoma and endocervical adenocarcinoma (CESC), Colon adenocarcinoma (COAD), Rectum adenocarcinoma (READ), Esophageal carcinoma (ESCA), Stomach and Esophageal carcinoma (STES), Thyroid carcinoma (THCA), Bladder Urothelial Carcinoma (BLCA) and Cholangiocarcinoma (CHOL) while downregulated in Lung adenocarcinoma (LUAD), Breast invasive carcinoma (BRCA), Kidney renal clear cell carcinoma (KIKP),Kidney renal papillary cell carcinoma (KIPAN),Prostate adenocarcinoma (PRAD),Head and Neck squamous cell carcinoma (NHSC) and Kidney Chromophobe (KICH) patients (Fig. 1A). In terms of OS, we observed a significant association between high expression of TGM1 and poor prognosis in several cancer types, including KIPAN, KIRC, Adrenocortical carcinoma (ACC), Skin Cutaneous Melanoma (SKCM), LIHC, and Pheochromocytoma and Paraganglioma (PCPG) (Fig. 1B). in terms of PFI, we observed a significant correlation between high expression of TGM1 and poor prognosis in patients with KIPAN, KIRC, ACC, STES, LIHC, Stomach adenocarcinoma (STAD), and BCLA and low expression of TGM1 was associated with poor prognosis in patients with GBMLGG and Thymoma (THYM) (Fig. 1C). The expression level of TGM1 in LUAD was found to be associated with the T stage, N stage, and M stage (Additional file 1: Fig.S1A–C). There are significant differences in TGM1 expression in different clinical stages and grades of Head and Neck squamous cell carcinoma (NHSC) (Additional file 1: Fig.S1D-F). Additionally, TGM1 expression levels in Lymphoid Neoplasm Diffuse Large B-cell Lymphoma (DLBC) and THYM are correlated with age (Additional file 1: Fig.S1G). In this study, we developed a prognostic nomogram that incorporates clinicopathological characteristics and TGM1 expression in patients with ACC, BLCA, and KIRC. The nomogram aims to predict the prognosis of these cancers based on these factors (Additional file 2: Fig.S2A-C).

In summary, we discovered several common cancer types and prognosis-related tumors that showed differential expression between tumor tissues and normal samples. The cancer types identified were ACC, KIKPAN, KIRC, and LIHC. Furthermore, our analysis revealed an age-related expression pattern of TGM1, with a positive correlation in THYM and a negative correlation in DLBC and GBMLGG.

### Relationship of TGM1 with tumor heterogeneity, stemness and gene mutation

**Fig.2:**
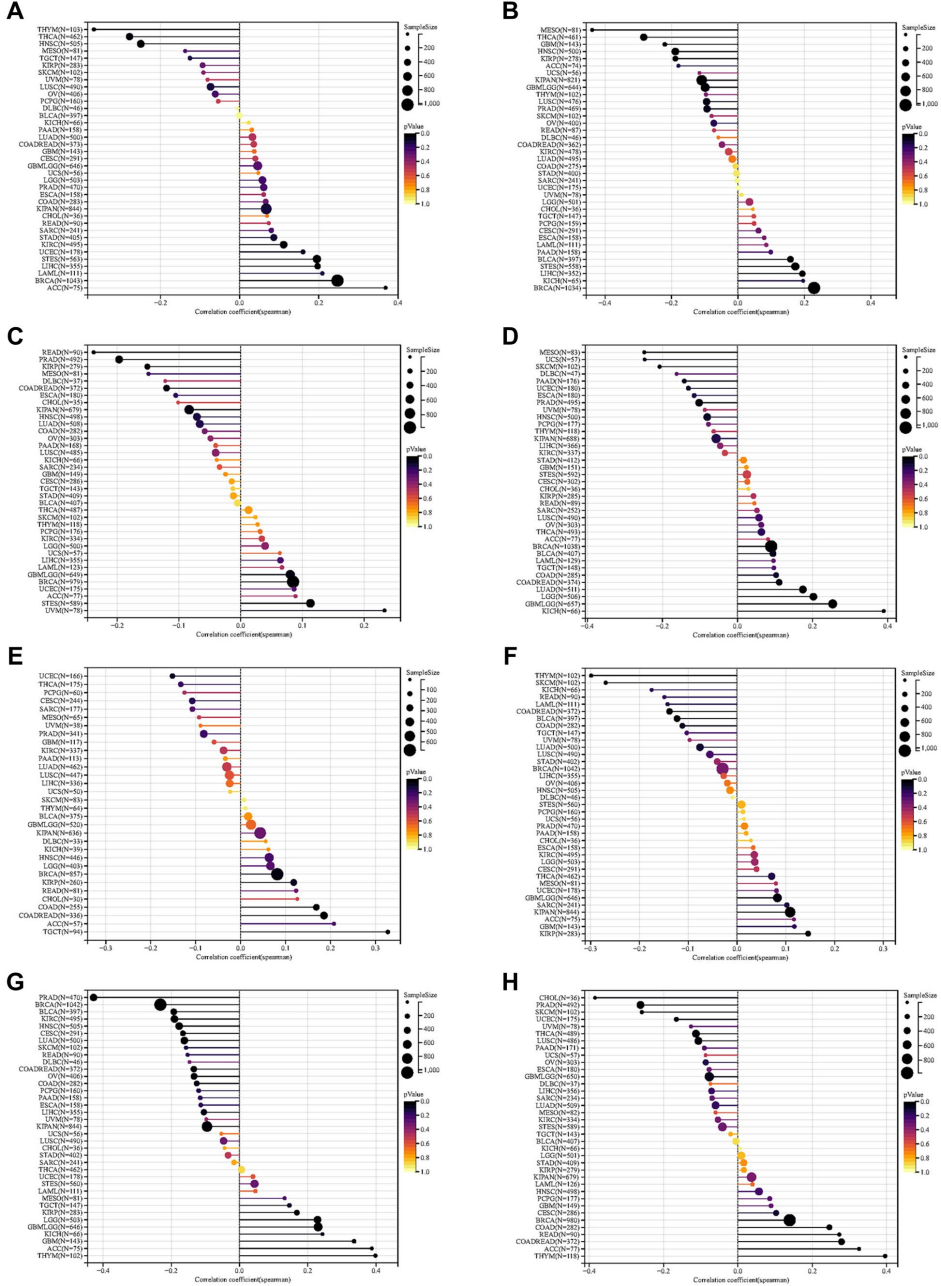
The pan-cancer Spearman analysis of tumor heterogeneity and TGM1 expression. **A** the correlation between HRD and TGM1 level; **B** the correlation between LOH and TGM1 level; **C** the correlation between MATH and TGM1 level; **D** the correlation between MSI and TGM1 level; **E** the correlation between NEO and TGM1 level; **(F)** the correlation between tumor ploidy and TGM1 level; **G** the correlation between tumor purity and TGM1 level; **H** the correlation between TMB and TGM1 level. HRD: homologous recombination deficiency; *LOH* loss of heterozygosity, *MATH*: mutant-allele tumor heterogeneity, *MSI* microsatellite instability, *NEO* neoantigen, *TMB* tumor mutation burden

**Fig.3:**
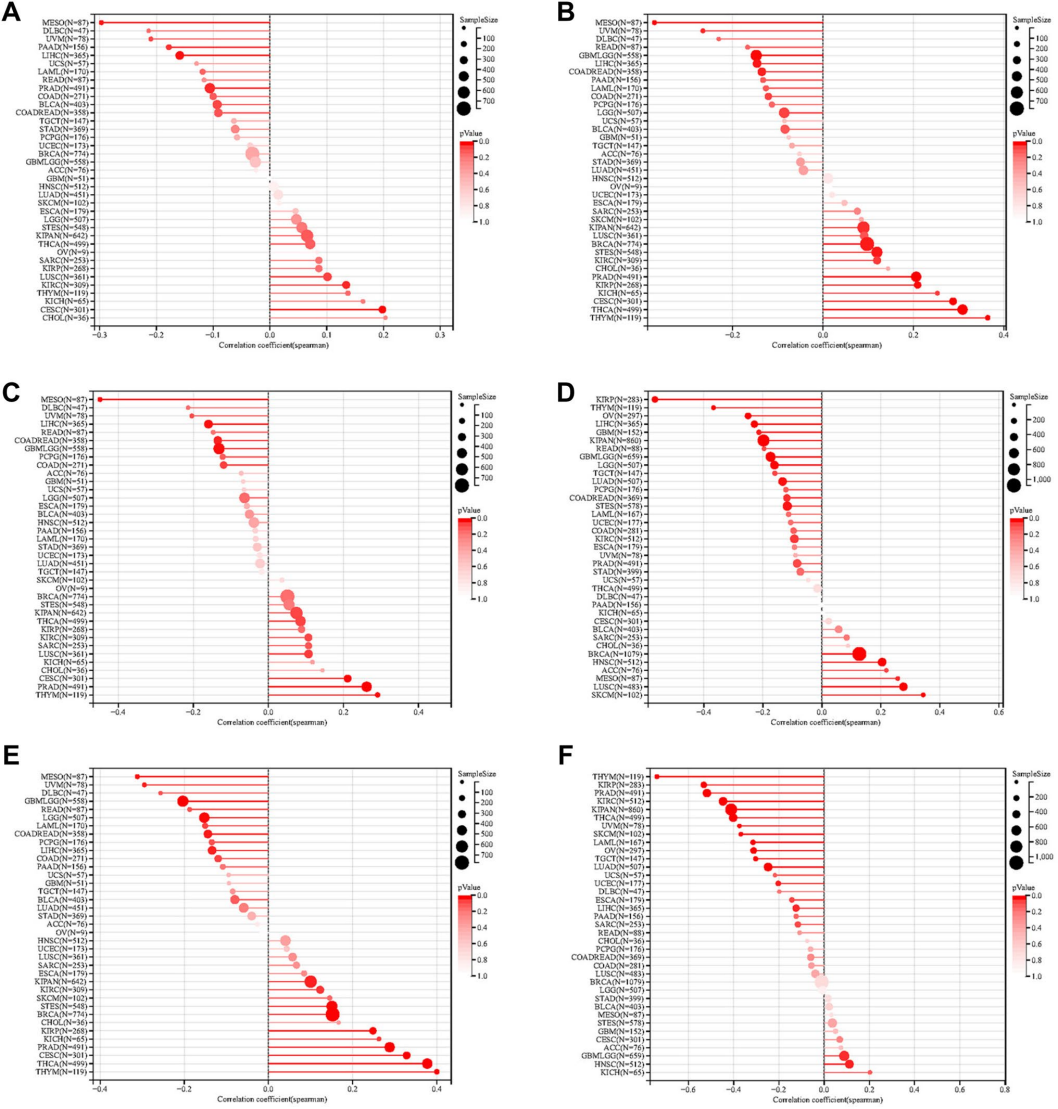
The pan-cancer Spearman analysis of tumor stemness and TGM1 expression. **A** The correlation between tumor stemness and TGM1 level using DMPss; **B** the correlation between tumor stemness and TGM1 level using DNAss; **C** the correlation between tumor stemness and TGM1 level using ENHss; **D** the correlation between tumor stemness and TGM1 level using EREG.EXPss; **E** the correlation between tumor stemness and TGM1 level using EREG-METHss; **F** the correlation between tumor stemness and TGM1 level using RNAss. *DNAss* DNA methylation based, *DMPss* differentially methylated probes-based, *EHNss* enhancer elements/DNA methylation-based, *RNAss* RNA expression-based, *EREG-METHss* epigenetically regulated DNA methylation-based, *EREG-METHss* epigenetically regulated RNA methylation-based

We further investigated the correlation between the expression level of TGM1 and tumor heterogeneity and stemness. Our study revealed significant correlations between TGM1 expression levels and HRD status in 14 tumors (Fig. 2A). Additionally, we observed a negative correlation between TGM1 expression and LOH in 9 tumors (Fig. 2B). In terms of MATH, we found a positive correlation between TGM1 mRNA expression and 4 tumors (Fig. 2C). MSI and NEO are indicators of tumor response to immunotherapy. Our findings demonstrated a significant correlation between TGM1 expression and MSI in 15 tumors, including HNSC, BRCA, and KICH. However, NEO was only correlated with TGM1 expression in 7 tumors (Fig. 2D, E). In our analysis of tumor stemness, we observed a negative correlation between the expression level of TGM1 in LIHC and all six tumor stemness (Fig. 3A–F).

**Fig.4:**
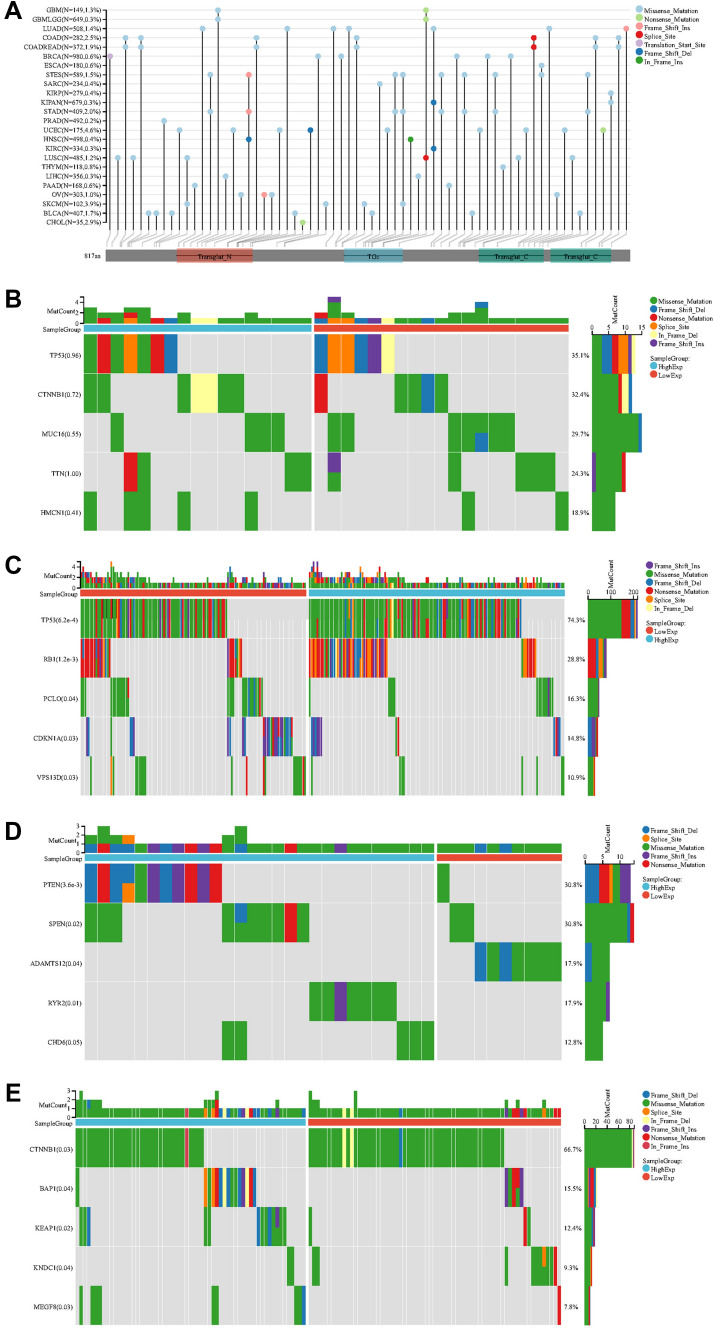
Mutation landscape of TGM1. **A** mutation landscapes of TGM1 for pan-cancer; **B** the top 5 mutation genes between high and low-expression of TGM1 in ACC patients; **C** the top 5 mutation genes between high and low-expression of TGM1 in BLCA patients; **D** the top 5 mutation genes between high and low-expression of TGM1 in KIRC patients; **E** the top 5 mutation genes between high and low-expression of TGM1 in LIHC patients

Some targeted therapeutic responses do not depend on tumor type, but rather on mutations. Tumor gene mutations are closely associated with their biological behavior. The study revealed mutation frequencies of 1.3% for GMB, 0.3% for KIRC, and 1.4% for LUAD (Fig. 4A). TGM1 was divided into high expression and low expression groups based on its expression level. The possible mechanism of TGM1 in tumorigenesis and development was explored through its mutated gene. No statistically significant gene mutations were found in ACC (Fig. 4B). In BLCA, significant gene mutations including TP53, RB1, PCLO, CDKN1A, and VPS13D were observed between the high expression group and low expression group (Fig. 4C). Likewise, KIRC showed significant gene mutations, such as PTEN, SPEN, ADAMTS12, RYR2, and CHD6, between the two groups (Fig. 4D). In LIHC, CTNNB1, BAP1, KEAP1, KNDC1, and MEGF8 were found to have significant differences in gene expression between the low and high expression groups (Fig. 4E).

### Relationship between TGM1 expression with immune regulation, checkpoints, RNA modification and drug sensitivity

**Fig.5:**
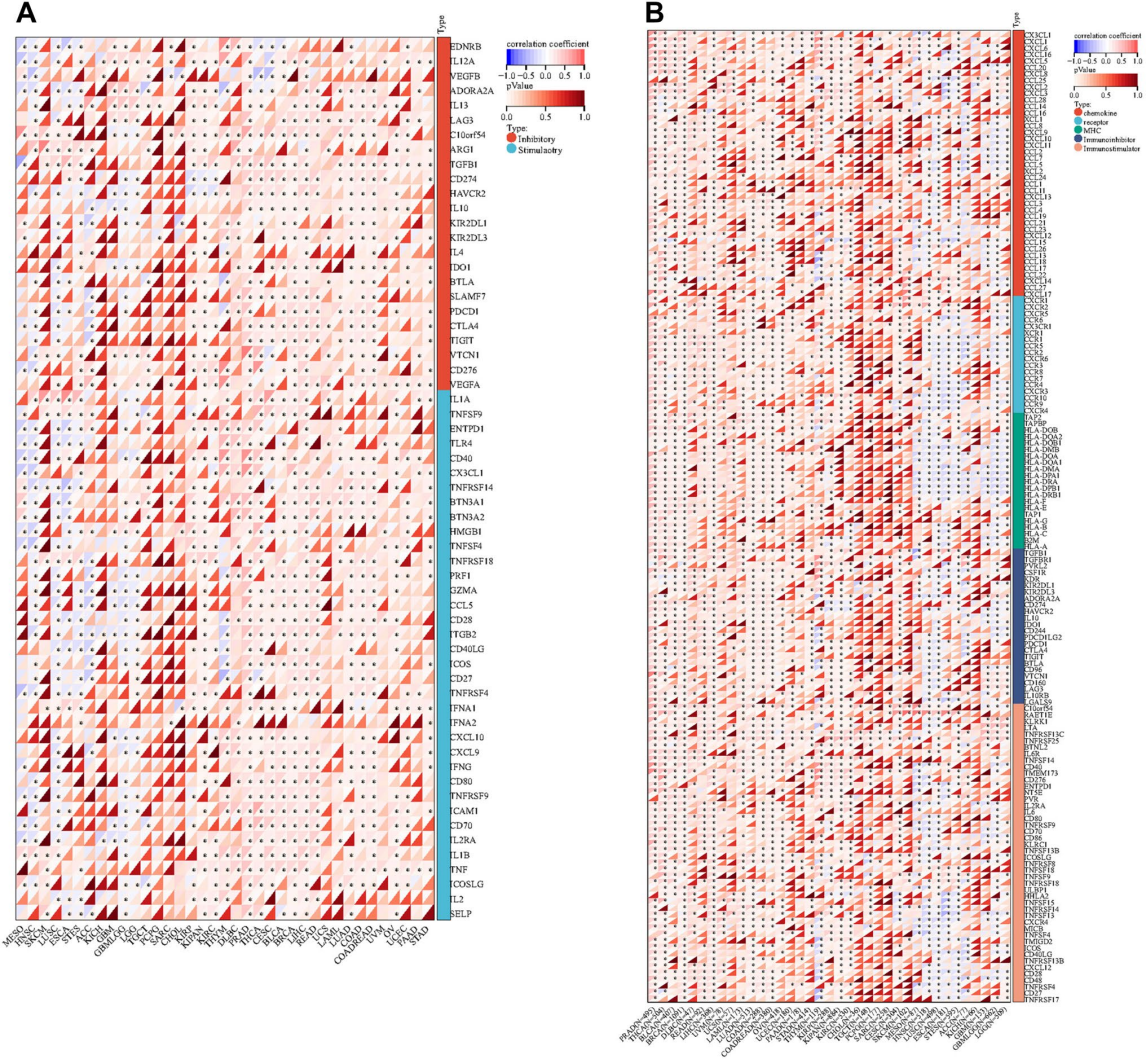
The Spearman analysis of TGM1 expression and regulatory genes and immune checkpoints. **A** the correlation of TGM1 expression with immune regulatory genes; **B** the correlation of TGM1 expression with immune checkpoint genes

**Fig.6:**
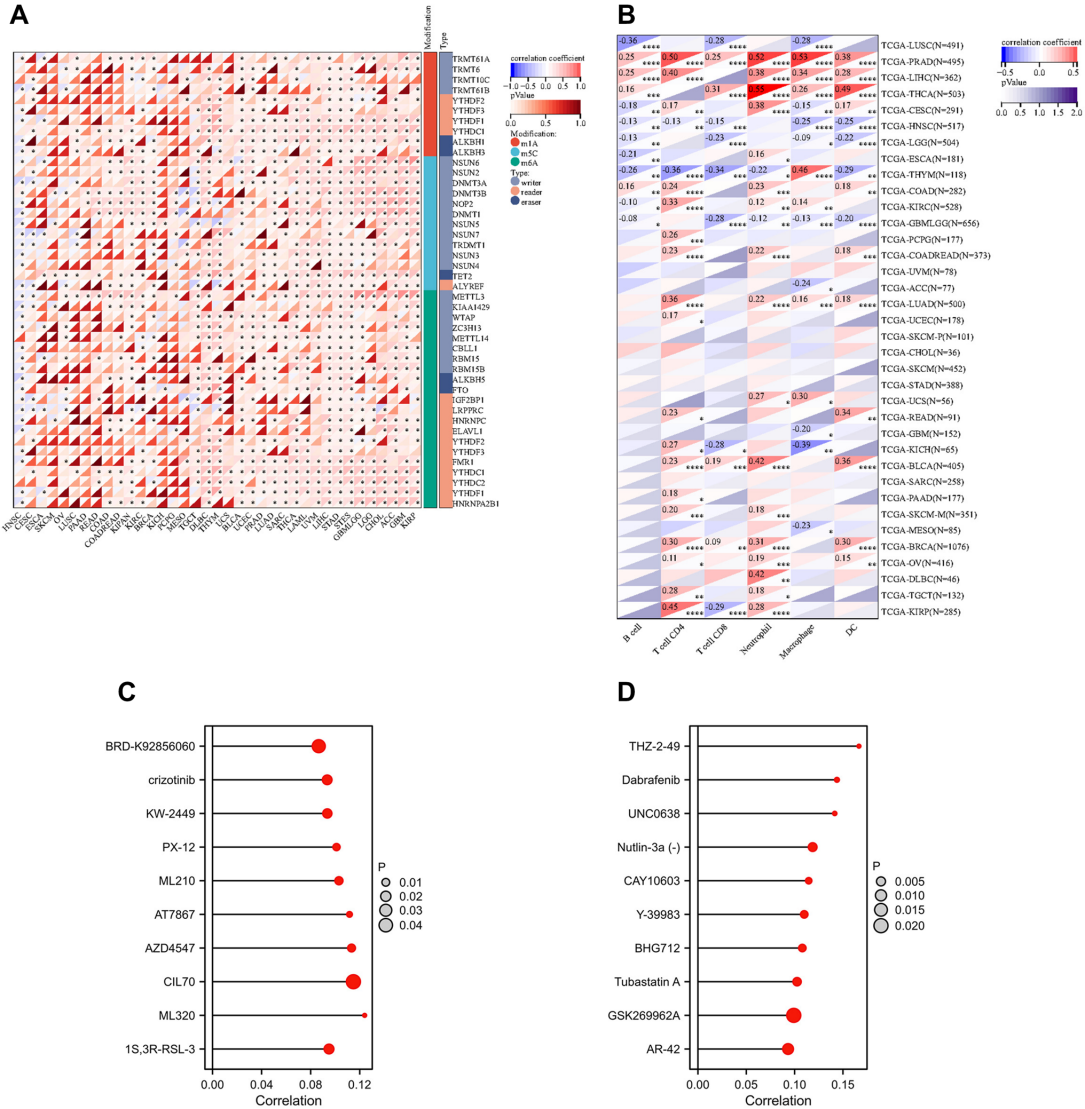
The Spearman analysis of TGM1 expression and RNA modification; Tumor immune environment and its correlation with TGM1 expression and drug sensitivity analysis. **A** the correlation of TGM1 expression with genes of RNA modification; **B** the correlation of TGM1 expression with immune infiltrating cells using TIMER; **C **the correlation between gene expression and the sensitivity of GDSC drugs (top 10) in pan-cancer; **D** the correlation between gene expression and the sensitivity of CTRP drugs (top 10) in pan-cancer

**Fig.7:**
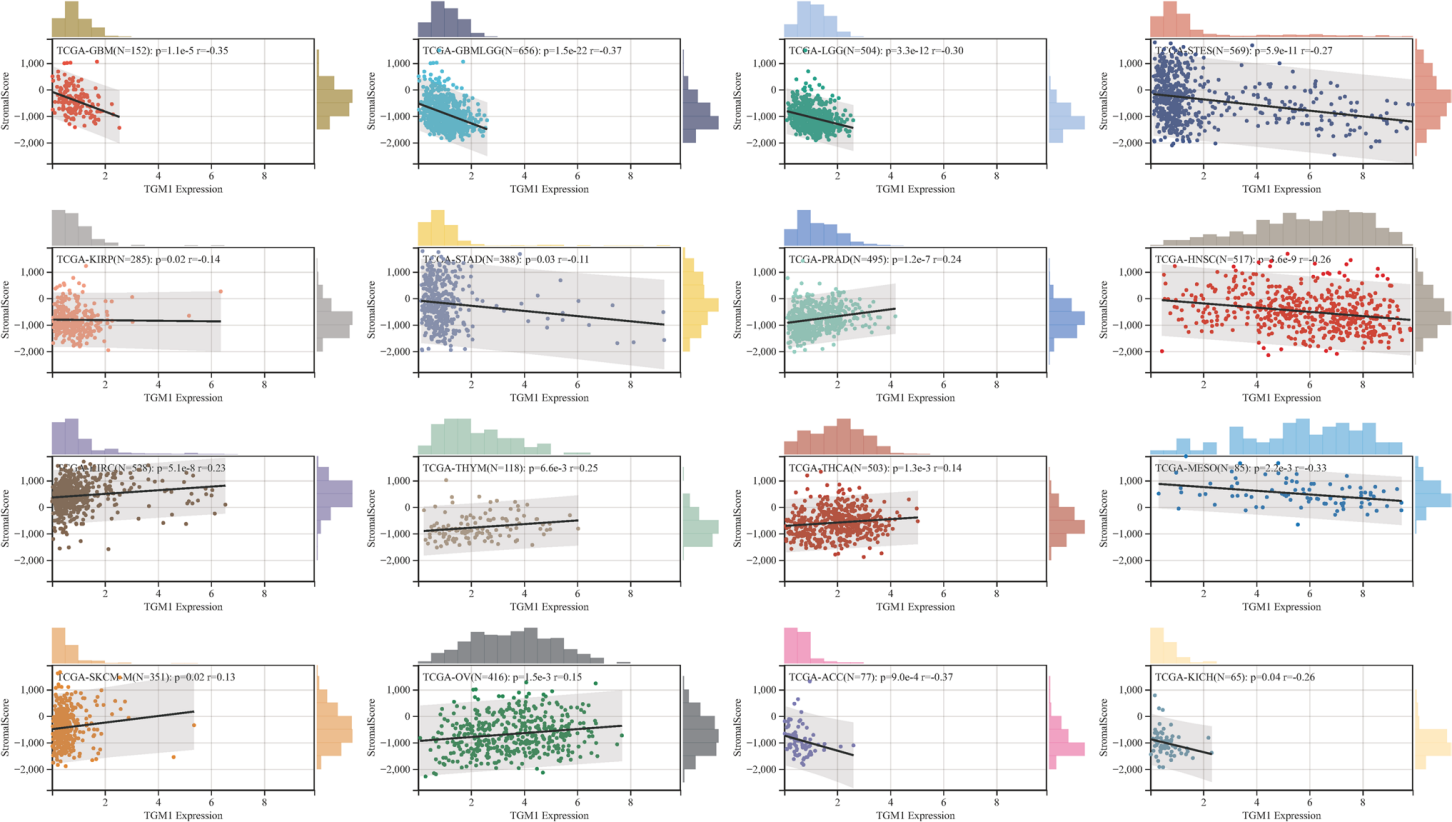
The correlation of TGM1 expression with stromal score

Our findings indicated that the expression levels of TGM1 in various cancer types were associated with multiple immune regulatory genes and immune checkpoint (IC) genes (Fig. 5A, B). Figure 5 demonstrates a correlation between the expression of TGM1 and various immune checkpoint genes and immunomodulatory genes in BLCA, LIHC, ACC, and even PRAD. However, it is worth noting that previous studies have indicated a limited effectiveness of immunotherapy in PRAD. In terms of RNA modification, our study revealed a negative correlation between TGM1 expression in ESCA and the expression of YTHDF1, LRPPRC, RBM15, ZC3H13, NSUN7, NSUN6, and TRMT61A (Fig. 6A). In the case of BLCA, we observed a positive correlation between TGM1 expression and multiple m1A, m5C, and m6A modifications (Fig. 6A). The results from Timer analysis demonstrated a positive correlation between TGM1 expression and the infiltration of several immune cell types in the tumor microenvironment (TME) of PRAD, LIHC, LUAD, and BRCA (Fig. 6B). For instance, in LIHC, TGM1 expression was positively correlated with the infiltration of B cells, CD4 + T cells, neutrophils, macrophages, and dendritic cells (Fig. 6B). We also observed an inverse correlation between TGM1 expression and macrophage infiltration in ACC (Fig. 6B). The correlation between TGM1 expression and drug sensitivity was analyzed in the pan-cancer analysis of GDSC and CTRP, as depicted in Fig. 6C and Fig. 6D, respectively. Among the tested drugs, Crizotinib, ML320, KW-2449, Dabrafenib, Tubastatin A, and CAY10603 demonstrated relatively favorable outcomes. We calculated Spearman's correlation coefficient between genes and immune infiltration scores in each tumor to determine the immune infiltration scores that showed significant correlation. Our analysis revealed that gene expression was significantly correlated with immune infiltration in 18 different cancer types. Among these, 7 cancer types showed a significant positive correlation, while 11 cancer types showed a significant negative correlation (Fig. 7).

## Discussion

Understanding the biological behavior of tumors and their treatment methods from multiple dimensions has always been a significant challenge due to the complex mechanism of tumor formation and development. The high mortality rate and treatment cost impose a significant burden on society (Carrera et al. 2018). However, with the advancements in high-throughput sequencing technology, we now have the ability to explore the genetic characteristics of tumors and identify new therapeutic targets (Song et al. 2023). Notably, tumor cells exhibit unlimited proliferation when cellular homeostasis is disrupted (Shen et al. 2022), making the gene TGM1, which is associated with cell proliferation and keratinization, a potential breakthrough in tumor therapy. TGM1 gene is composed of 15 exons and is located on chromosome 14q11.2 (Polakowska et al. 1991). It is a protein-coding gene that encodes the TGase-1 enzyme, which has a molecular weight of 89 kDa and consists of 817 amino acids (Hu et al. 2019). Its primary function is to catalyze the cross-linking of proteins and the combination of polyamines and proteins, playing a role in cell proliferation (Zhang et al. 2016). Previous studies have linked TGM1 to autosomal recessive lamellar ichthyosis and non-bullous congenital ichthyosis erythroderma (Farasat et al. 2009; Kawashima et al. 2005; Landegren et al. 2021). While there is limited research on TGM1 in tumors, Ding et al. (2021) have shown that TGM1 is upregulated in ascites-derived ovarian cancer cells, potentially contributing to metastasis and spread. In vitro experiments have also demonstrated a relationship between TGM1 and cell proliferation in gastric cancer cells (Huang et al. 2017).

In this study, we conducted a bioinformatic analysis of patient data from each dataset to assess the expression of TGM1 in tissues. Our findings revealed that TGM1 mRNA levels were significantly upregulated in 12 tumors, including bladder cancer, when compared to their corresponding normal tissues. These results are consistent with previous studies conducted by Huang et al. (2017), which also reported an upregulation of TGM1 in gastric cancer cells compared to normal tissues. Furthermore, the study suggested that knockdown of TGM1 could potentially enhance apoptosis. This has also been confirmed in a study on Alzheimer's disease. Tripthy et al. (2020) observed an increase in the expression of TGM1 in the hippocampus and primary cortical neurons of a mouse model during the late stage of the disease, leading to cell death. Based on these findings, it is hypothesized that the progression of TGM1 in tumors might be associated with apoptosis. However, further experiments are required to validate this hypothesis. We also investigated the prognostic value of TGM1 by analyzing tumor OS and PFI. However, the exact role of TGM1 in predicting prognosis remains unclear. In our study, we observed that TGM1 was associated with poor prognosis in some tumors, but interestingly, it acted as a protective factor in tumors like GBMLGG. Our findings showed that the expression level of TGM1 was correlated with the poor prognosis of SKCM patients, a malignancy that mainly occurs in the elderly (Long et al. 2022). This aligns with a study by Li et al. (2021), where they discovered that TGM1 was significantly down-regulated in metastatic SKCM tissues compared to primary SKCM tissues, suggesting potential involvement of TGM1 in the occurrence and spread of SKCM tumors. We hypothesize that the differences in TGM1 expression levels and prognosis could be attributed to epigenetic regulation. We investigated the correlation between TGM1 expression and clinical features, specifically TNM stages. Our findings revealed that TGM1 expression levels varied across different T stages, N stages, and M stages of LUAD. This suggests that the expression of TGM1 may be influenced by epigenetic regulation triggered by heterogeneity during tumor development or metastasis. Furthermore, our results indicate that the role of TGM1 may differ at different stages of various tumors, making it a potential predictor for clinical staging and guiding therapy. Our study also demonstrates a correlation between age and TGM1 expression. Aging is a well-known risk factor for various tumors, as it results in reduced cell proliferation, homing, and differentiation processes (Feng et al. 2023; Hu et al. 2022; Feng et al. 2020). These abnormalities in the human body contribute to the promotion and development of tumors (Ruiz et al. 2023). Hence, it is crucial to identify the genetic intersection between aging and tumors and subsequently intervene in this process.

Tumors consist of a diverse population of cells with high heterogeneity, which can be categorized into intra-tumor heterogeneity and inter-tumor heterogeneity (Dagogo-Jack and Shaw 2018). This heterogeneity arises from variations in genetics, epigenetics, and the tumor microenvironment (Chu et al. 2022; Liu et al. 2023a). Understanding tumor heterogeneity is crucial for studying tumor growth, metastasis, apoptosis and drug resistance, and it holds significant promise for identifying potential therapeutic approaches. We conducted an analysis of 8 indicators that reflect tumor heterogeneity, including TMB and MSI. TMB measures the number of genomic mutations, while the number of neoantigens expressed in tumor cells increases in proportion to the mutational burden (Jardim et al. 2021). This increase in neoantigens enhances the likelihood of positive responses to checkpoint blockade immunotherapy, making it a potential biomarker for anti-PD-1/PD-L1 therapy (Goodman et al. 2017; Joshi and Durden 2019). Previous studies have demonstrated that melanoma patients with a high mutational burden experienced improved survival following ipilimumab treatment (Gupta et al. 2015). Additionally, TMB has been found to predict the survival rate of non-small cell lung cancer patients undergoing immunotherapy (Hellmann et al. 2022). Our study discovered a significant association between the expression of TGM1 and TMB in 14 tumors. This finding indicates that the expression level of TGM1 has an impact on tumor heterogeneity, resulting in alterations in TMB. Consequently, these changes may influence the response of patients to immunotherapy. Two major forms of genomic instability commonly found in tumors are chromosomal instability and MSI (Ogino and Goel 2008). MSI-high tumors exhibit defects in the mismatch repair system, resulting in phenotypic hypermutation and the generation of immunogenic neoantigens (Grasso et al. 2018). This frequently leads to a significant infiltration of lymphocytes in these tumors, which is associated with favorable clinical outcomes (Ge et al. 2023; Pei et al. 2023). Our study demonstrated a positive correlation between the expression level of TGM1 and MSI in seven tumors, including COAD and BLCA. Previous studies have also indicated the prognostic value of high MSI in these two tumor types (Awadalla et al. 2022; Lochhead et al. 2013). Therefore, our results may serve as a valuable reference for the selection of immunotherapy approaches.

Through genome sequencing of tumor samples from cancer patients, the relative abundance of gene mutations has been determined for various forms of cancer (Xu et al. 1986). Identifying commonly mutated genes is crucial for drug development, as some oncoproteins can be targeted by drugs (Bollag et al. 2010; Loriot et al. 2019). This knowledge can help allocate public resources to benefit a larger number of patients. In this study, we aim to investigate the role of TGM1 in tumors by analyzing gene mutations based on its expression levels. Our findings reveal that PTEN mutation is the most common in KICH, and it exerts an anti-tumor effect by inhibiting the activation of PI3K/AKT (Peglion et al. 2022). Voss et al. (2019) conducted a study on patients with advanced KICH who were treated with everolimus and sunitinib. The study found that patients with positive PTEN protein expression had a better prognosis when treated with everolimus. However, no significant difference in prognosis was observed in patients treated with sunitinib. These findings could potentially assist clinicians in making informed decisions regarding treatment options for patients with advanced KICH. Moreover, PTEN is expressed in prostate cancer cell lines PC3 and DU145, which are androgen-independent and represent advanced stages of the disease (Brussel et al. 1999; Huang et al. 2001; Calastretti et al. 2014). PTEN mutation may can serve as a marker to predict treatment response and the developmental stage of cancer cells. Previous studies have demonstrated that TP53 mutations can result in the inactivation of wild-type p53 and contribute to tumor progression (Kotler, et al. 2018; Hu et al. 2021). In addition to TP53 mutations, our findings reveal a higher mutation frequency in RB1 in BLCA. Importantly, a study conducted by Liu et al. (2023b) observed that BLCA tissues with co-mutations in TP53 and RB1 exhibited a greater presence of immune effectors, which showed a significant correlation with the response to immune checkpoint inhibitors (ICIs). Mutated KEAP1 has a higher mutation frequency in the LIHC group with low TGM1 expression, and KEAP1 mutation is associated with poor prognosis in many tumors (Romero et al. 2020). Our findings revealed a positive correlation between the expression level of TGM1 and several RNA modification genes, including m1A, m5C, and m6A. Notably, this correlation was particularly evident in LIHC, KIRC, BRCA, CHOL, and ACC. Previous studies have partially explored the prognostic significance of m6A modification in BRCA and CHOL (Feng et al. 2022c; Wei et al. 2023). Furthermore, m5C can serve as a potential biomarker for predicting the efficacy of LIHC immunotherapy (Liu et al. 2022).

Our findings indicate that the expression of TGM1 plays a crucial role in tumor immunity within the TME. TME encompasses various components, including immune cells, cytokines, and oxidative stress-related products, which collectively influence tumor growth and survival (Xiao and Yu 2021; Xiong et al. 2023a). To investigate the relationship between TGM1 expression and tumor-infiltrating immune cells in the TME, we utilized the TIMER method to evaluate the infiltration score of different cell types. Our results revealed a positive correlation between TGM1 expression and CD4 + T cells as well as neutrophils in most tumors. CD4 + T cells play a vital role in enhancing the intratumoral cytotoxic T lymphocyte response through signal transduction, thereby promoting the effectiveness of immunotherapy (Borst et al. 2018). Additionally, neutrophils exhibit tumor-killing capabilities by releasing catalytically active elastase (Cui, et al. 2021). Furthermore, TGM1 expression showed a positive correlation with multiple immune regulatory genes, suggesting that its high expression level may indicate increased sensitivity to immunotherapy. In addition, immune checkpoint genes have the ability to influence immune cell function. IC signaling is activated as tumors progress, enabling immune evasion (Feng et al. 2019). Therefore, examining the relationship between TGM1 expression and immune checkpoint markers can offer fresh opportunities for the development of innovative immunosuppressants. We conducted an analysis on 60 immune checkpoint genes and found that TGM1 expression exhibited a positive correlation with immune checkpoints in the majority of tumors (Feng et al. 2023b). This suggests the potential of TGM1 as a viable drug target. Medicines are undoubtedly the most important resource in the treatment of cancer. Our analysis revealed a correlation between the sensitivity of multiple drugs and the expression of TGM1. Crizotinib, a multi-target protease inhibitor, is commonly used in tumor patients with abnormal AKL, ROS, and MET kinase activities (Blackhall and Cappuzzo 2016). It has demonstrated promising therapeutic effects in tumors (Shaw et al. 2020; Pal et al. 2021). Based on our study, we propose that TGM1 could serve as a potential target gene of crizotinib for clinical application.

Our study reveals that the expression of TGM1 in BLCA is associated with a poor prognosis. However, the mechanism of TGM1 in BLCA remains unexplored. Our previous study have indicated that TGM1 may utilize exogenous metabolic pathways, such as cytochrome P450 and steroid hormone biosynthesis, to contribute to the development and progression of tumors (Wang et al. 2023). Additionally, Huang et al. discovered that TGM1 activates the Wnt signaling pathway, promoting the development of gastric cancer (Huang et al. 2017). The Wnt/β-catenin signaling pathway regulates mesenchymal changes by controlling the levels of cell adhesion molecules. It is possible that this pathway also plays a role in tumor epithelial-mesenchymal transition, but its involvement in bladder cancer cells is yet to be determined. Further investigations are required to verify this hypothesis.

Like BLRC, KIRC is a prevalent malignant tumor in urinary system. Among the different pathological types of renal cancer, KIRC is the most observed. Our study revealed that the expression of TGM1 in normal tissues of KIRC is higher compared to tumor tissues. Moreover, a high expression of TGM1 is associated with a poor prognosis. These findings suggest that TGM1 may not act as a tumor suppressor in KIRC. However, its expression shows a strong correlation with immune checkpoints and has the potential to be a therapeutic target. Further research is still required to validate these findings.

Our study demonstrates that the role of TGM1 in tumors is not limited to a single tumor type. We examined the correlation between TGM1 expression and prognosis, clinical characteristics, tumor heterogeneity, gene mutation, and immune infiltration in pan-cancer patients. This investigation sheds light on the role of TGM1 in tumorigenesis and development, and comprehensively evaluates its potential as a prognostic marker and drug target from various perspectives. However, it is important to acknowledge the limitations of our work. Firstly, the heterogeneity of different databases may slightly impact the reliability of our analysis results. Moreover, our findings are based on public database analysis and encompass multiple tumor types, necessitating further experiments to elucidate the mechanism of TGM1. Nevertheless, our pan-cancer analysis of TGM1 provides a solid foundation and novel insights for future research.

## Conclusion

TGM1 has the potential to serve as both a prognostic marker and a drug target.

## Supplementary Information

Below is the link to the electronic supplementary material.

Supplementary file1: Fig. S1. The correlation of TGM1 expression with Clinicopathological features **A** The correlation of TGM1 expression with gender; **B** The correlation of TGM1 expression with grade; **C** The correlation of TGM1 expression with clinical stages; **D** The correlation of TGM1 expression with T stages; **E** the correlation of TGM1 expression with N stage; **F** The correlation of TGM1 expression with M stages; **G** The correlation of TGM1 expression with age(TIF 2139 KB)

Supplementary file2: Fig. S2. Nomogram of TGM1 expression and tumor pathological characteristics. **A** Nomogram of ACC; **B** Nomogram of BLCA; **C** Nomogram of KICH. *ACC* Adrenocortical carcinoma, *BLCA* Bladder Urothelial Carcinoma, *KICH* Kidney Chromophobe(TIF 2301 KB)

## Data Availability

The data sets presented in this study are available in online repositories. The name and join number of the repository can be found in the article/supplement.

## Abbreviations

ACC: Adrenocortical carcinoma
BLCA: Bladder Urothelial Carcinoma
BRCA: Breast invasive carcinoma
CESC: Cervical squamous cell carcinoma and endocervical adenocarcinoma
CHOL: Cholangiocarcinoma
COAD: Colon adenocarcinoma
READ: Rectum adenocarcinoma Esophageal carcinoma
DLBC: Lymphoid Neoplasm Diffuse Large B-cell Lymphoma
ESCA: Esophagus carcinoma
GBM: Glioblastoma multiforme
GBMLGG: Glioma
HNSC: Head and Neck squamous cell carcinoma
KICH: Kidney Chromophobe
KIPAN: Pan-kidney cohort (KICH + KIRC + KIRP)
KIRC: Kidney renal clear cell carcinoma
KIRP: Kidney renal papillary cell carcinoma
LAML: Acute Myeloid Leukemia
LGG: Brain Lower Grade Glioma
LIHC: Liver hepatocellular carcinoma
LUAD: Lung adenocarcinoma
LUSC: Lung squamous cell carcinoma
MESO: Mesothelioma
OV: Ovarian serous cystadenocarcinoma
PAAD: Pancreatic adenocarcinoma
PCPG: Pheochromocytoma and Paraganglioma
PRAD: Prostate adenocarcinoma
READ: Rectum adenocarcinoma
SARC: Sarcoma
STAD: Stomach adenocarcinoma
SKCM: Skin Cutaneous Melanoma
STES: Stomach and Esophageal carcinoma
TGCT: Testicular Germ Cell Tumors
THCA: Thyroid carcinoma
THYM: Thymoma
UCEC: Uterine Corpus Endometrial Carcinoma
UCS: Uterine Carcinosarcoma
UVM: Uveal Melanoma
TARGET-OS: Osteosarcoma
TARGET-ALL: Acute Lymphoblastic Leukemia
TARGET-NB: Neuroblastoma
TARGET-WT: High-Risk Wilms Tumor
TGM2: Transglutaminase type 2
TGM1: Transglutaminase type 1
TCGA: The Cancer Genome Atlas
OS: Overall survival
PFI: Progression-free interval
DNAss: DNA methylation based
DMPss: Differentially methylated probes-based
EHNss: Enhancer elements/DNA methylation-based
RNAss: RNA expression-based
EREG-METHss: Epigenetically regulated DNA methylation-based
EREG-METHss: Epigenetically regulated RNA methylation-based
TMB: Tumor mutation burden
MATH: Mutant-allele tumor heterogeneity
LOH: Loss of heterozygosity
NEO: Neoantigen
HRD: Homologous recombination deficiency
MSI: Microsatellite instability
IC: Immune checkpoint
TME: Tumor microenvironment
GDSC: Genomics of Drug Sensitivity in Cancer
CTRP: Cancer Therapeutics Response Portal
