# Supplementary figures and images for: A pan-cancer analysis of the oncogenic and immunological roles of transglutaminase 1 (TGM1) in human cancer
Source: J Cancer Res Clin Oncol. 2024 Mar 12;150(3):123. doi: 10.1007/s00432-024-05640-6 (PMC10933153; doi:10.1007/s00432-024-05640-6)

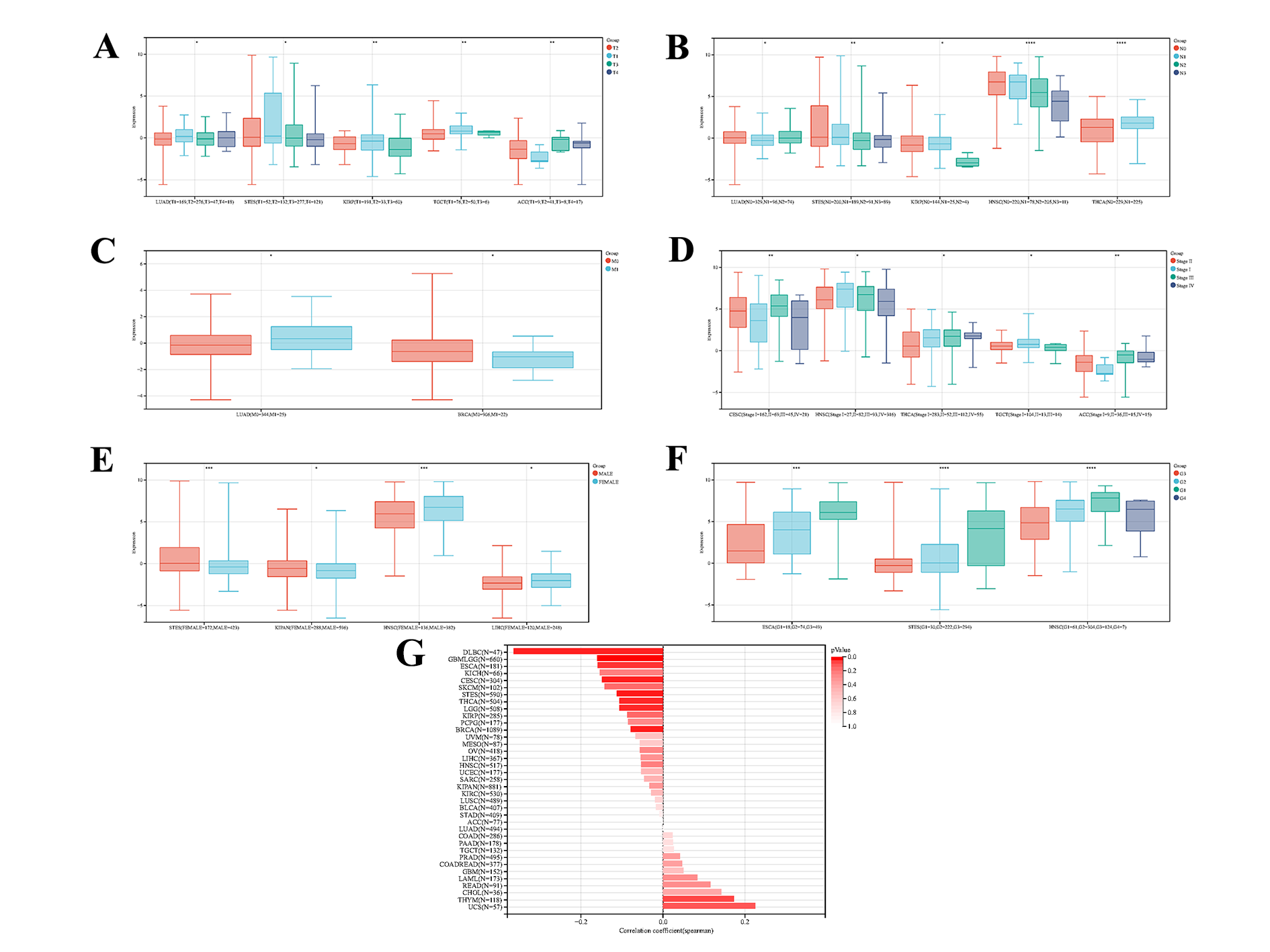

Supplement: Supplementary file 1 — Supplementary file1: Fig. S1. The correlation of TGM1 expression with Clinicopathological features A The correlation of TGM1 expression with gender; B The correlation of TGM1 expression with grade; C The correlation of TGM1 expression with clinical stages; D The correlation of TGM1 expression with T stages; E the correlation of TGM1 expression with N stage; F The correlation of TGM1 expression with M stages; G The correlation of TGM1 expression with age(TIF 2139 KB) [file 432_2024_5640_MOESM1_ESM.tif]

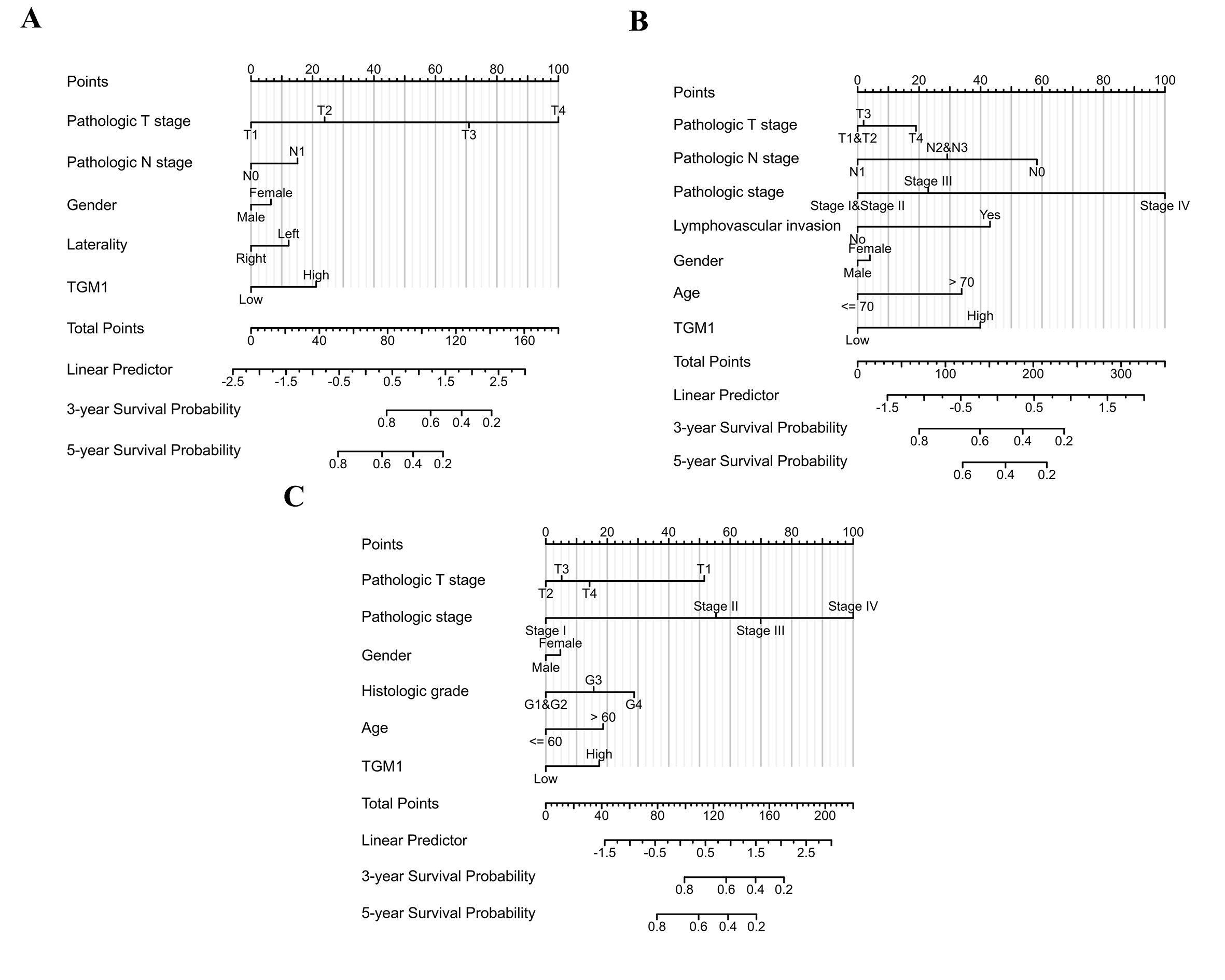

Supplement: Supplementary file 2 — Supplementary file2: Fig. S2. Nomogram of TGM1 expression and tumor pathological characteristics. A Nomogram of ACC; B Nomogram of BLCA; C Nomogram of KICH. ACC Adrenocortical carcinoma, BLCA Bladder Urothelial Carcinoma, KICH Kidney Chromophobe(TIF 2301 KB) [file 432_2024_5640_MOESM2_ESM.tif]
